# Cumulative trauma load and timing of trauma prior to military deployment differentially influences inhibitory control processing across deployment

**DOI:** 10.1038/s41598-023-48505-7

**Published:** 2023-12-05

**Authors:** Lisa N. Miller, David Forbes, Alexander C. McFarlane, Ellie Lawrence-Wood, Julian G. Simmons, Kim Felmingham

**Affiliations:** 1 Melbourne School of Psychological Science, Trauma Anxiety and Stress Lab, The University of Melbourne, Level 7, Redmond Barry Building, Melbourne, VIC 3010 Australia; 2 Department of Psychiatry, The University of Melbourne, Melbourne, Australia; 3 Phoenix Australia, Centre for Posttraumatic Mental Health, Melbourne, Australia; 4 Discipline of Psychiatry, Adelaide Medical School, University of Adelaide, Adelaide, South Australia Australia

**Keywords:** Cognitive neuroscience, Stress and resilience, Psychology

## Abstract

Military personnel experience high trauma load that can change brain circuitry leading to impaired inhibitory control and posttraumatic stress disorder (PTSD). Inhibitory control processing may be particularly vulnerable to developmental and interpersonal trauma. This study examines the differential role of cumulative pre-deployment trauma and timing of trauma on inhibitory control using the Go/NoGo paradigm in a military population. The Go/NoGo paradigm was administered to 166 predominately male army combat personnel at pre- and post-deployment. Linear mixed models analyze cumulative trauma, trauma onset, and post-deployment PTSD symptoms on NoGo-N2 and NoGo-P3 amplitude and latency across deployment. Here we report, NoGo-N2 amplitude increases and NoGo-P3 amplitude and latency decreases in those with high prior interpersonal trauma across deployment. Increases in NoGo-P3 amplitude following adolescent-onset trauma and NoGo-P3 latency following childhood-onset and adolescent-onset trauma are seen across deployment. Arousal symptoms positively correlated with conflict monitoring. Our findings support the cumulative trauma load and sensitive period of trauma exposure models for inhibitory control processing in a military population. High cumulative interpersonal trauma impacts conflict monitoring and response suppression and increases PTSD symptoms whereas developmental trauma differentially impacts response suppression. This research highlights the need for tailored strategies for strengthening inhibitory control, and that consider timing and type of trauma in military personnel.

## Introduction

Trauma exposure may be a key etiological factor for psychopathology found in military populations. Military personnel are exposed to higher levels of trauma than the general population, with deployment-related trauma, particularly combat, and trauma prior to military service, particularly developmental trauma, being associated with increased psychopathology, such as posttraumatic stress disorder (PTSD)^1–3^. Trauma exposure may compromise the inhibitory control system increasing PTSD through increased demand on conflict monitoring due to difficulty regulating threat processing and reactivity, and impaired response inhibition due to difficulty suppressing salient trauma-related stimuli during or after deployment^4–6^.

Trauma exposure may impair inhibitory control processing through several mechanisms. One of the original theories for PTSD is the cumulative stress model, which proposes the accumulation of traumatic events leads to allostatic load where brain circuitry is overstimulated or impaired, thus increasing risk of PTSD^7,8^. Research supports this theory showing individuals exposed to at least four different trauma types have a higher risk of impulsivity and PTSD, with a greater vulnerability following interpersonal, but not non-interpersonal, trauma as well as early-onset trauma^9–13^. This suggests the timing of the trauma, as well as cumulative interpersonal trauma, may be another important factor to consider. Trauma exposure at different ages differentially impacts psychopathology in adulthood^14^. Previous research shows cumulative developmental (child and adolescent) interpersonal trauma is associated with impaired inhibitory control and may be a stronger predictor of PTSD than adult interpersonal trauma^15^. A more recent theory for PTSD is the sensitive period of trauma exposure model, which suggests enhanced threat detection and response following developmental trauma may be an adaptive circuitry change to avoid threat, however by altering trajectories of brain development involved in threat detection, emotion regulation and inhibitory control it may increase PTSD when facing trauma in adulthood^16,17^.

Inhibitory control processing involves conflict monitoring, the detection and control of competing responses, and response suppression, the ability to suppress an activated response^18^. The Go/NoGo paradigm can be used to study inhibitory control by measuring conflict monitoring of Go and NoGo stimuli, and response suppression to NoGo stimuli^18–20^. Event related potentials (ERPs), averaged transient electrical potentials, can be used to discern high temporal resolution information processing and cortical function from the Go/NoGo paradigm. The two ERPs most associated with the Go/NoGo paradigm are the NoGo-N2 reflecting conflict monitoring and the NoGo-P3 reflecting response suppression^18,19,21,22^. Go/NoGo ERP studies with source localization most consistently observed the NoGo-N2 in the anterior cingulate cortex (ACC), and the NoGo-P3 in the pre-frontal regions including the orbitofrontal cortex, inferior-frontal cortex and supplementary motor cortex^19,23–25^. Meta-analytic review revealed veterans with PTSD display enhanced resources for conflict monitoring (larger NoGo-N2 amplitude) and delayed response inhibition (longer P3 latency), and police with sub-clinical PTSD displaying enhanced resources for response inhibition (larger P3 amplitude)^22,25–28^. Trauma-exposed adolescents with PTSD displayed faster conflict monitoring (shorter NoGo-N2 latency) suggesting impulsivity may be related to faster monitoring or detection of conflict following trauma in adolescence^28^. Healthy adults with high developmental (childhood and adolescent) trauma also show increased impulsivity due to hypoactivation in the ACC and medial prefrontal cortex to the NoGo-P3^29^. This suggests impaired inhibitory control in adults, regardless of PTSD, may be related to trauma at critical developmental periods.

Inhibitory control processing in the brain develops differentially with age. Bottom-up attention processing develops before age 10, thus trauma in childhood may impair conflict monitoring and the NoGo-N2 which peaks in amplitude at age six and then decreases with age^18,30^. The inhibitory control system in the pre-frontal region begins developing around age 10 and increases connectivity to the amygdala and ACC to enhance conflict monitoring and emotion regulation^30–35^. The NoGo-P3 amplitude appears around age 10 and increases with age, suggesting it may be vulnerable to trauma in adolescence^18,30^. These inhibitory frontal connections appear altered following brain injury^36^, trauma at critical developmental periods^17,37^ as well as in PTSD^38–41^. Therefore conflict monitoring and NoGo-N2, and response inhibition and NoGo-P3 develop differentially, and childhood and adolescence onset trauma may produce a vulnerability to PTSD through disrupted development of these prefrontal brain structures and inhibitory connectivity with the limbic and memory systems leading to impaired inhibitory control processing in adulthood^14,32,33^.

To date no ERP studies have examined the impact of cumulative trauma nor timing of trauma using a Go/NoGo paradigm from a cross-sectional nor longitudinal perspective. Further, most studies examining ‘childhood trauma’ define this as a trauma before age 18. Therefore, the aim of this study is to examine the impact of cumulative trauma (interpersonal, non-interpersonal), trauma onset (childhood, adolescent, and adulthood) and PTSD symptoms on ERPs using a Go/NoGo paradigm at pre- and post-deployment. Firstly, we hypothesize trauma-onset will explain more variance in inhibitory control processing than cumulative trauma types. Secondly, as interpersonal trauma is more commonly associated with PTSD than non-interpersonal trauma, we hypothesize higher interpersonal, but not non-interpersonal, trauma load will result in enhanced conflict monitoring and delayed response inhibition across deployment. Based on previous developmental (child/adolescent) research, we predict child/adolescent trauma will be associated with impaired conflict monitoring and response inhibition across deployment compared to the adult-onset and no trauma groups. As previous studies have not separated child-onset from adolescent-onset trauma, we will perform an exploratory analysis looking at differences between trauma-onset groups, and the relationship of PTSD symptoms on the predictive patterns of cumulative and timing of trauma.

## Results

Table 1 provides participant demographics, trauma exposure and PTSD symptoms. Refer to Supplementary Materials for full statistical comparison of demographics by trauma onset. Participants were predominately males aged in their late twenties. The ANOVA and post-hoc t-test showed the adult-onset trauma group was significantly older than the no trauma (*p* < 0.001) and adolescent-onset (*p* = 0.010) groups, and had significantly more times deployed and higher combat exposure than other trauma-onset groups (Times Deployed: child *p* = 0.006, adolescent *p* < 0.001, no trauma *p* < 0.001; Combat: child *p* = 0.005, adolescent *p* = 0.014; no trauma *p* < 0.001). The adolescent-onset trauma group had significantly more times deployed than the no trauma group (*p* = 0.046). The ANOVA and post-hoc t-test of cumulative trauma types (CTT), particularly interpersonal CTT, showed this was significantly higher in the childhood-onset than the adolescent-onset and adult-onset trauma groups (Overall: *p* = 0.042 and *p* = 0.003 respectively; and Interpersonal: *p* = 0.014 and *p* = 0.004 respectively).

**Table 1:** Participant demographics, trauma exposure and PTSD symptoms by Trauma onset.

	Overall	Childhood trauma	Adolescent trauma	Adult trauma	No prior trauma	p-value
N	166	20	45	57	44	
Pre-deployment						
Male	96%	95%	98%	96%	95%	0.891
Age	28.8 (7.06)	28.5 (6.32)	28.24 (6.44)	31.72 (7.16)	25.49 (6.45)	< 0.001
Times deployed	2.93 (3.53)	2.56 (2.64)	2.26 (2.46)	4.96 (4.42)	0.79 (1.08)	< 0.001
Trauma types	2.57 (2.46)	4.75 (2.69)	3.56 (1.99)	3.02 (2.07)	0 (0)	0.010
Interpersonal	2.04 (2.16)	4.00 (2.64)	2.64 (1.82)	2.44 (1.91)	0 (0)	0.012
Combat	34%	40%	29%	61%		
Sexual abuse	2%	10%	2%	0%		
Emotional abuse	4%	30%	0%	2%		
Physical abuse	39%	70%	73%	30%		
Found body	28%	45%	33%	40%		
Domestic violence	6%	20%	7%	5%		
Witnessed trauma	55%	80%	78%	70%		
Non-interpersonal	0.54 (0.68)	0.75 (0.72)	0.91 (0.70)	0.58 (0.68)	0 (0)	0.059
Natural disaster	27%	50%	44%	26%		
Life-threatening	27%	25%	47%	32%		
PCL	19.67 (5.04)	20.4 (5.46)	20.4 (5.40)	19.96 (5.76)	18.03 (2.38)	0.126
Re-experiencing	5.55 (1.31)	5.8 (1.88)	5.73 (1.21)	5.58 (1.39)	5.15 (0.81)	0.156
Avoidance	8.06 (2.39)	8.7 (2.52)	8.24 (2.77)	8.19 (2.66)	7.31 (0.83)	0.124
Arousal	6.07 (1.97)	5.9 (1.52)	6.42 (2.17)	6.19 (2.3)	5.56 (1.23)	0.225
Post-deployment						
PCL	23.09 (9.24)	21.35 (4.3)	24.63 (9.28)	22.1 (7.68)	23.44 (12.12)	0.508
Re-experiencing	6.52 (2.62)	6.06 (1.34)	6.80 (2.53)	6.23 (1.74)	6.77 (3.8)	0.587
Avoidance	9.12 (3.92)	8.29 (2.17)	9.41 (3.37)	8.81 (3.59)	9.54 (5.26)	0.636
Arousal	7.46 (3.37)	7.00 (1.87)	8.41 (3.93)	7.06 (3.00)	7.13 (3.59)	0.201
Combat exposure	35.31 (16.99)	30.59 (16.74)	34.9 (15.91)	43.47 (15.67)	27.95 (15.98)	< 0.001
High PTSD	14%	6%	24%	8%	13%	0.135
mTBI	39%	44%	47%	44%	22%	0.102

*PCL* Post-traumatic stress disorder symptoms, *mTBI* Mild Traumatic Brain Injury.

All values refer to mean (standard deviation), unless indicated otherwise as percentage.

P-values are from ANOVA for continuous variables and Fisher’s test for categorical variables.

### Model fit comparison

Table 2 provides the model fit comparison from the F-test based on the Kenward-Roger’s approach by ERP component. For N2 amplitude and latency, there was a significant improvement by adding the interaction of Deployment with Interpersonal and Non-interpersonal CTT. There was no improvement by adding the Deployment*Trauma-onset interaction nor a 3-way interaction with PTSD. For P3 amplitude and latency, there was a significant improvement by adding the interaction of Deployment*Interpersonal CTT and Deployment*Non-interpersonal CTT, and Deployment*Trauma-onset. There was no improvement with adding a 3-way interaction for PTSD.

**Table 2:** Results from the F-test assessing model fit comparison for N2 and P3 Component.

Model	Marginal R^2^ (%)	△Marginal R^2^ (%)	F (DF)	p-value
N2 amplitude				
Baseline	5.34			
+ CTT	6.73	1.39	2.90 (4, 258)	0.023
+ TrOnset	9.88	3.15	1.88 (6, 268)	0.084
+ PTSD	10.76	0.89	0.84 (12, 279)	0.609
N2 latency				
Baseline	7.93			
+ CTT	10.22	2.28	4.10 (4, 258)	0.003
+ TrOnset	10.80	0.59	1.02 (6, 268)	0.410
+ PTSD	11.46	0.65	0.68 (12, 279)	0.768
P3 amplitude				
Baseline	3.94			
+ CTT	6.05	2.11	4.88 (4, 292)	0.001
+ TrOnset	8.22	2.18	2.64 (6, 304)	0.016
+ PTSD	8.54	0.31	0.50 (12, 316)	0.916
P3 latency				
Baseline	4.14			
+ CTT	5.16	1.01	3.39 (4, 291)	0.010
+ TrOnset	6.38	1.22	2.37 (6, 303)	0.030
+ PTSD	7.85	1.47	0.65 (12, 313)	0.802

Baseline model: ERP ~ mTBI + Age + Sex + Combat Exposure + Site + Deployment + (1/StudyID/Site), CTT = Cumulative Interpersonal and non-interpersonal Trauma Type (as 2 separate summed variables), TrOnset = Trauma Onset (4gp: Trauma < 10, Trauma 10–17, Trauma 18 + , No Trauma), Post-deployment PTSD = PCL Score (2gp: PCL < 30 Low; PCL >  = 30 High), F (DF) = F value (Num DF, Den DF).

### N2 component

The linear mixed model showed significant predictors of N2 amplitude were Site, Deployment, and 2-way interactions of Deployment*Interpersonal CTT and Deployment*Non-interpersonal CTT (Table 3). The fixed effects explained 7% of the variance in N2 Amplitude. Supplementary Materials provides a full breakdown of post-hoc analyses.

**Table 3:** Omnibus Test for N2 Amplitude and Latency.

	Sum Sq	Mean Sq	F (DF)	p-value
Amplitude				
mTBI	13.84	13.84	1.91 (1, 131)	0.169
Age	0.23	0.23	0.03 (1, 131)	0.859
Sex	14.22	14.22	1.97 (1, 129)	0.163
Combat exposure	1.44	1.44	0.20 (1, 130)	0.656
Site	80.84	80.84	11.18 (1, 386)	0.001
Deployment	106.39	106.39	14.71 (1, 389)	< 0.001
CTT_InT	6.99	6.99	0.97 (1, 131)	0.327
CTT_NInT	6.71	6.71	0.93 (1, 131)	0.337
Deployment:CTT_InT	41.22	41.22	5.70 (1, 394)	0.017
Deployment:CTT_NInT	50.40	50.40	6.97 (1, 391)	0.009
Latency				
mTBI	63.25	63.25	0.28 (1, 132)	0.600
Age	2209.15	2209.15	9.66 (1, 131)	0.002
Sex	259.37	259.37	1.13 (1, 128)	0.289
Combat exposure	228.38	228.38	1.00 (1, 130)	0.319
Site	619.98	619.98	2.71 (1, 386)	0.100
Deployment	1243.07	1243.07	5.44 (1, 390)	0.020
CTT_InT	294.54	294.54	1.29 (1, 132)	0.258
CTT_NInT	503.17	503.17	2.20 (1, 131)	0.140
Deployment:CTT_InT	189.59	189.59	0.83 (1, 396)	0.363
Deployment:CTT_NInT	3050.41	3050.41	13.34 (1, 392)	< 0.001

*F (DF)* F-value (Num DF, Den DF), *mTBI* mild traumatic brain injury, *CTT_InT* cumulative interpersonal trauma type, *CTT_NInT* cumulative non-interpersonal trauma type.

**Figure 1:**
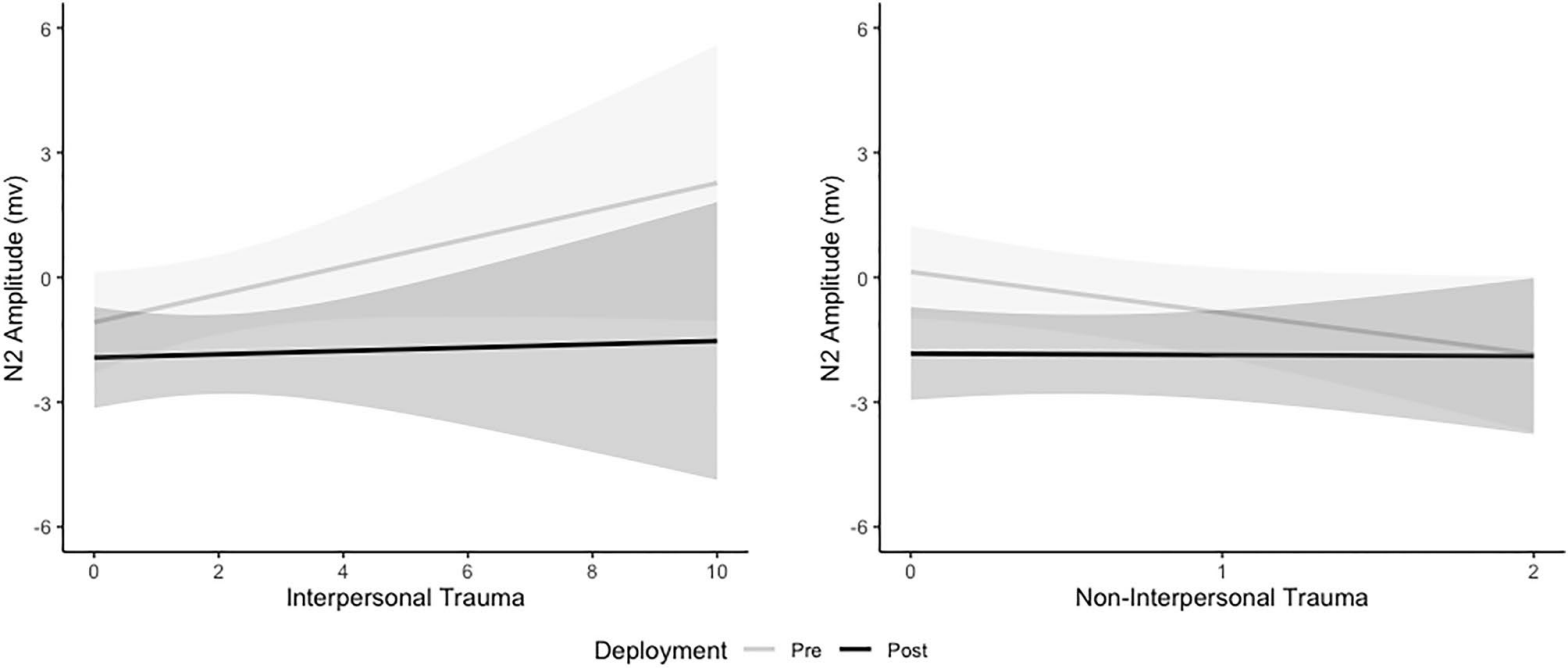
Estimated Marginal Trends from the linear mixed model with 95% Confidence Intervals in N2 Amplitude by Interpersonal and Non-interpersonal Cumulative Trauma Type across Deployment. N2 amplitude is a negative ERP component so smaller values indicate larger magnitude.

After accounting for other variables in the linear mixed model, the main effect of Site had a significant and small effect on N2 amplitude, where amplitude at FCz was 0.78mV greater than Cz (b =  − 0.78, SE = 0.22, *p* = 0.001, Cohen’s d = 0.17). The Deployment*Interpersonal CTT interaction (Fig. 1) shows that after accounting for other variables in the linear mixed model, change in N2 amplitude with increasing interpersonal CTT was larger at pre-deployment compared to post-deployment and the effect was small (b =  − 0.30mV, *p* = 0.018, Cohen’s d = 0.06). N2 amplitude at pre-deployment decreased by 0.34mV, 95%CI (− 0.11, 0.79) with every 1 additional interpersonal CTT with little change at post-deployment (b =  − 0.04mv, 95%CI (− 0.42, 0.50)). The Deployment*non-interpersonal CTT interaction (Fig. 1) shows that after accounting for other variables in the linear mixed model, change in N2 amplitude with non-interpersonal CTT was significantly larger at pre-deployment than post-deployment and the effect was small (b =  − 0.96mV, *p* = 0.009, Cohen’s d =  − 0.20). N2 amplitude at pre-deployment increased by 0.99mV, 95%CI (− 2.24, 0.27) with every 1 trauma increase in non-interpersonal CTT with little change at post-deployment (b = -0.03mV, 95%CI (− 1.30, 1.24)).

**Figure 2:**
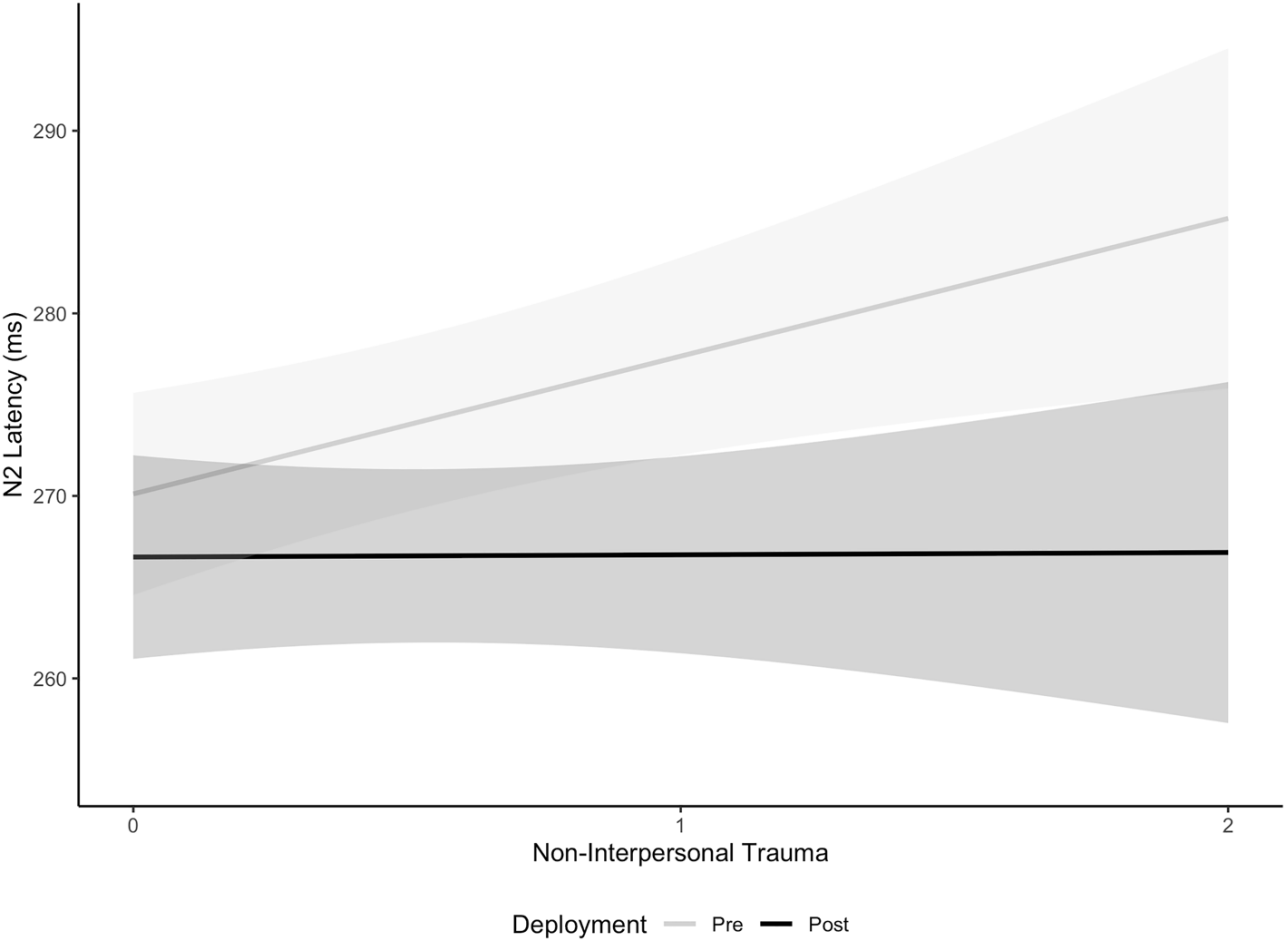
Estimated Marginal Trends (with 95% Confidence Intervals) from the linear mixed model for N2 Latency by Non-interpersonal Cumulative Trauma Type across Deployment.

The linear mixed model for N2 latency showed significant predictors were Age, Deployment, and Deployment*Non-interpersonal CTT interaction (Table 3). Supplementary Materials provides a full breakdown of post-hoc analyses. After accounting for other variables in the linear mixed model, the main effect of Age had a significant yet small effect on N2 latency, with N2 latency increasing by 0.82ms with every one-year increase in Age at pre-deployment (b = 0.82, SE = 0.26, *p* = 0.002, Cohen’s d = 0.03). The Deployment*Non-interpersonal CTT interaction (Fig. 2) shows that after accounting for other variables, change in N2 latency with increasing non-interpersonal CTT was significantly larger at pre-deployment compared to post-deployment and the effect was small (b = 7.42, *p* < 0.001, Cohen’s d = 0.31). N2 latency at pre-deployment increased by 7.55ms, 95%CI (1.30, 13.79) with every 1 trauma increase in non-interpersonal CTT with little change at post-deployment (b = 0.12ms, 95% CI (− 6.19, 6.40)).

### P3 component

The linear mixed model for P3 amplitude showed a significant main effect of Site, and Non-interpersonal CTT, and 2-way interactions of Deployment*Interpersonal CTT and Deployment*Non-interpersonal CTT, and Deployment*Trauma-onset (Table 4). The fixed effects explained 8% of the variance in P3 Amplitude. Supplementary Materials provides a full breakdown of post-hoc analyses.

**Table 4:** Omnibus Test for the P3 Amplitude and Latency.

	Sum Sq	Mean Sq	F (DF)	p-value
Amplitude				
mTBI	2.26	2.26	0.31 (1, 128)	0.576
Age	9.28	9.28	1.29 (1, 129)	0.258
Sex	1.81	1.81	0.25 (1, 126)	0.617
Combat exposure	4.95	4.95	0.69 (1, 127)	0.408
Site	294.10	147.05	20.47 (2, 269)	< 0.001
TrOnset	27.90	9.30	1.30 (3, 128)	0.279
Deployment	0.83	0.83	0.12 (1, 388)	0.734
CTT_InT	9.86	9.86	1.37 (1, 128)	0.244
CTT_NInT	32.99	32.99	4.59 (1, 128)	0.034
Deployment:TrOnset	87.42	29.14	4.06 (3, 389)	0.007
Deployment:CTT_InT	57.11	57.11	7.95 (1, 391)	0.005
Deployment:CTT_NInT	31.86	31.86	4.44 (1, 389)	0.036
Latency				
mTBI	768.00	768.00	2.95 (1, 128)	0.088
Age	274.40	274.40	1.05 (1, 130)	0.306
Sex	4.70	4.70	0.02 (1, 126)	0.893
Combat exposure	87.60	87.60	0.34 (1, 126)	0.563
Site	6261.80	3130.90	12.04 (2, 644)	< 0.001
TrOnset	269.50	89.80	0.35 (3, 128)	0.793
Deployment	7166.30	7166.30	27.55 (1, 648)	< 0.001
CTT_InT	65.20	65.20	0.25 (1, 128)	0.617
CTT_NInT	3.40	3.40	0.01 (1, 128)	0.909
Deployment:TrOnset	3504.00	1168.00	4.49 (3, 651)	0.004
Deployment:CTT_InT	1558.80	1558.80	5.99 (1, 652)	0.015
Deployment:CTT_NInT	1615.80	1615.80	6.21 (1, 652)	0.013

*F (DF)* F-value (Num DF, Den DF), *mTBI* mild traumatic brain injury, *CTT_InT* cumulative interpersonal trauma type, *CTT_NInT* cumulative non-interpersonal trauma type, *TrOnset* trauma onset (4gp: Trauma < 10, Trauma 10–17, Trauma 18 + , No Trauma).

After accounting for other variables in the linear mixed model, the main effect of Site had a significant and small effect on P3 amplitude, where amplitude at Cz was 1.51mV greater than at Fz (b = 1.51, SE = 0.24, *p* < 0.001, Cohen’s d = 0.36) and 0.89mV greater than at Pz (b = 0.89, SE = 0.24, *p* = 0.001, Cohen’s d = 0.21) and Pz was 0.62mV greater than at Fz (b = 0.62, SE = 0.24, *p* = 0.029, Cohen’s d = 0.15).

**Figure 3:**
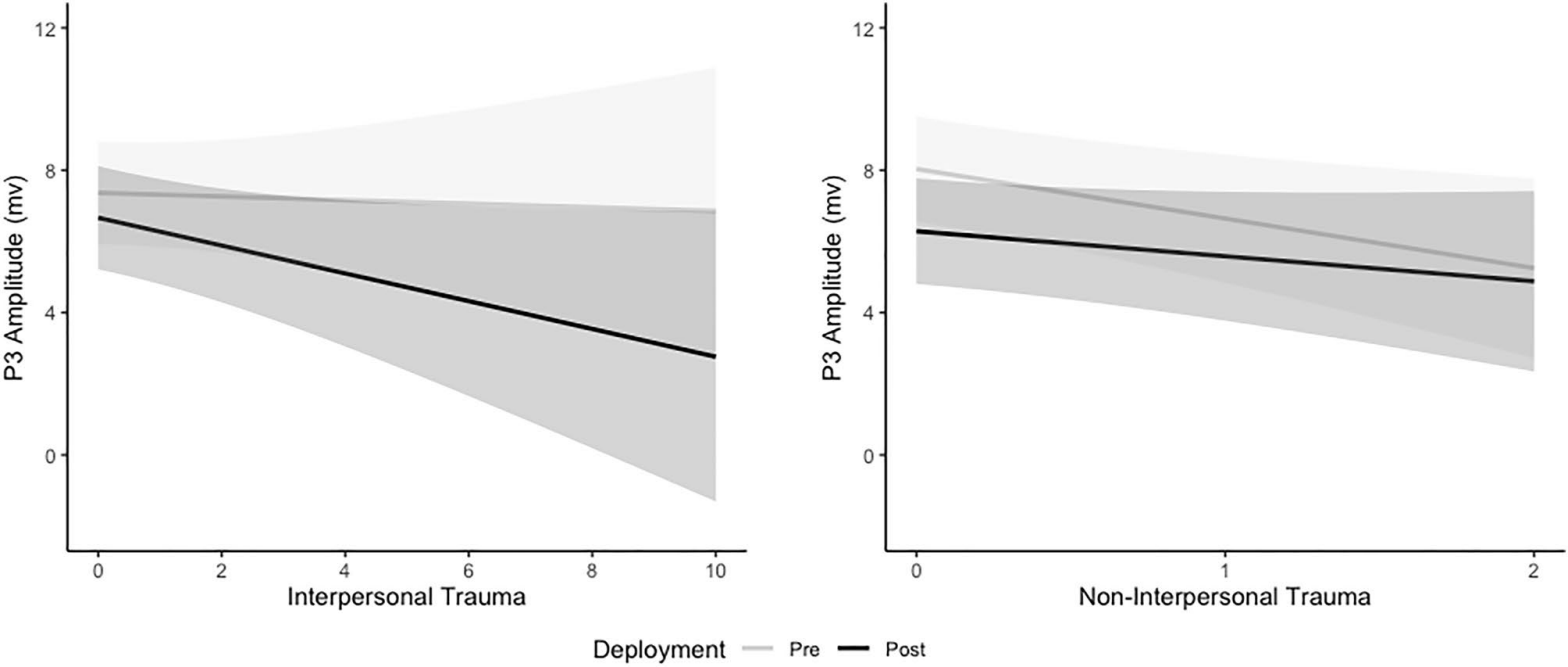
Estimated Marginal Trends (with 95% confidence intervals) from the linear mixed model for P3 Amplitude by Interpersonal and Non-interpersonal Cumulative Trauma Type across deployment.

The Deployment*Interpersonal CTT interaction (Fig. 3) shows that after accounting for other variables in the linear mixed model, change in P3 amplitude with increasing interpersonal CTT was larger at post-deployment compared to pre-deployment and the effect was small (b = 0.34, *p* = 0.005, Cohen’s d = 0.08). P3 amplitude at pre-deployment decreased by 0.05mV, 95%CI (− 0.50, 0.39) with every 1 additional Interpersonal CTT compared to 0.39mV, 95%CI (− 0.84, 0.06) at post-deployment. The Deployment*Non-interpersonal CTT interaction (Fig. 3) shows that after accounting for other variables in the linear mixed model, change in P3 amplitude with increasing non-interpersonal CTT was significantly larger at pre-deployment compared to post-deployment and the effect was small (b =  − 0.69, *p* = 0.036, Cohen’s d =  − 0.17). P3 amplitude at pre-deployment decreased by 1.39mV, 95%CI (− 0.2.55, − 0.23) with every 1 trauma increase in non-interpersonal CTT compared to 0.70mV, 95% CI (− 1.88, 0.47) at post-deployment.

**Figure 4:**
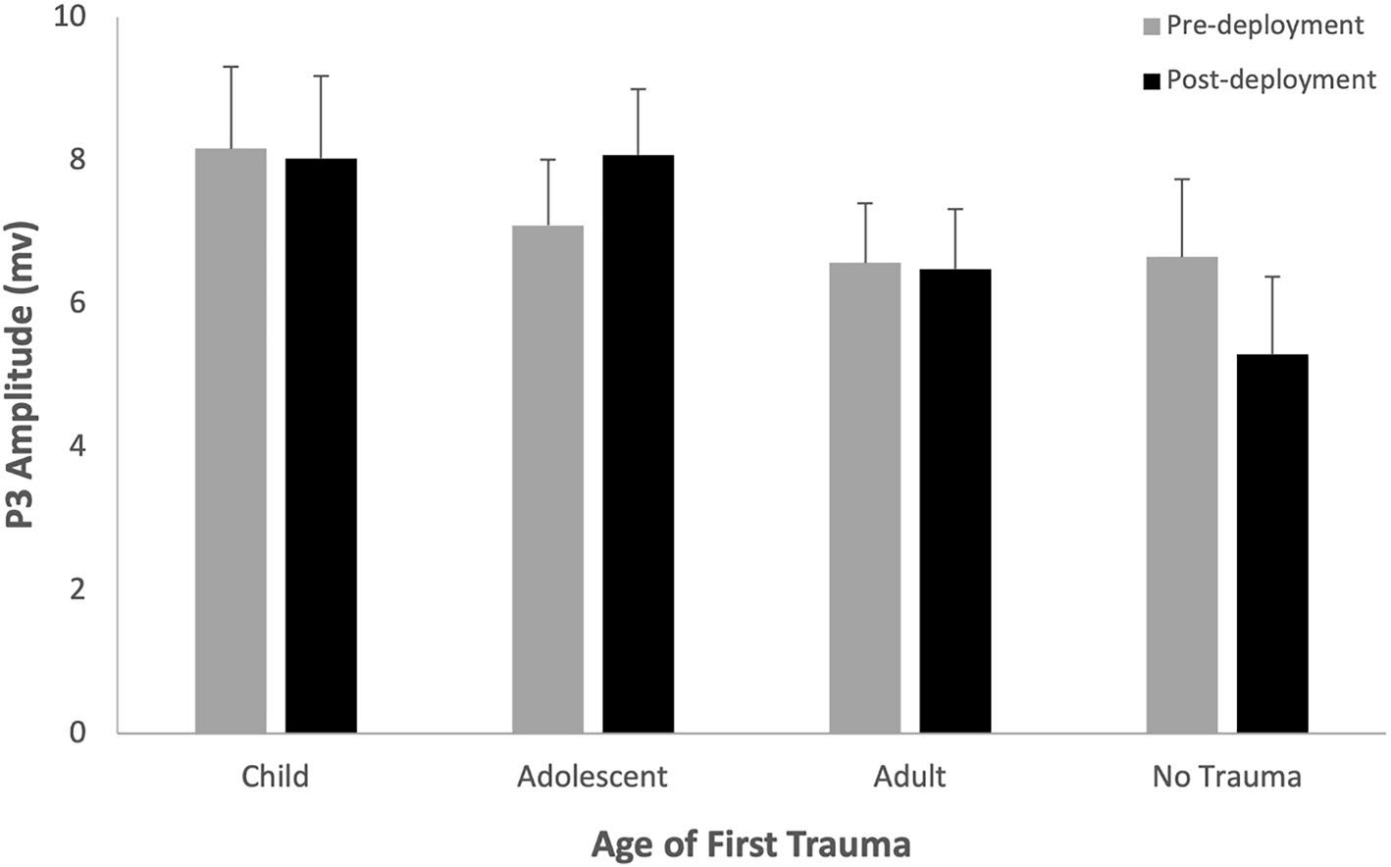
Estimated Marginal Means (with Standard Error) from the linear mixed model for P3 Amplitude by Trauma Onset across Deployment.

The Deployment*Trauma-Onset interaction (Fig. 4) shows that after accounting for other variables in the linear mixed model, there was a significant decrease in P3 amplitude for the no trauma group (b = 1.37, SE = 0.49, *p* = 0.006) and significant increase in P3 amplitude for the adolescent-onset group (b = -0.97, SE = 0.39, *p* = 0.013). Change in P3 amplitude across deployment was greater in the no trauma group compared to the adolescent-onset (b = 2.34, SE = 0.70, *p* = 0.001) and adult-onset (b = 1.28, SE = 0.60, *p* = 0.034) groups, and adolescent-onset was greater than the adult-onset group (b = 1.06, SE = 0.51, *p* = 0.037). There was no difference in P3 amplitude between trauma groups at pre- or post-deployment (refer to Supplementary Materials).

The linear mixed model for P3 Latency showed a significant main effect of Site, Deployment, and 2-way interactions of Deployment*Interpersonal CTT, Deployment*Non-interpersonal CTT and Deployment*Trauma-Onset. The fixed effects explained 6% of the variance in P3 latency (Table 4). Supplementary Materials provides a full breakdown of post-hoc analyses.

After accounting for other variables in the linear mixed model, the main effect of Site had a significant and large effect on P3 latency, where latency at Pz was 6.60ms slower than at Fz (b = 6.60, SE = 1.42, *p* < 0.001, Cohen’s d = 1.59) and 0.5.24ms slower than at Cz (b = 5.24, SE = 1.41, *p* = 0.001, Cohen’s d = 1.26). There was no difference in P3 latency between Cz and Fz (b =  − 1.36, SE = 1.40, *p* = 0.999, Cohen’s d =  − 0.33).

**Figure 5:**
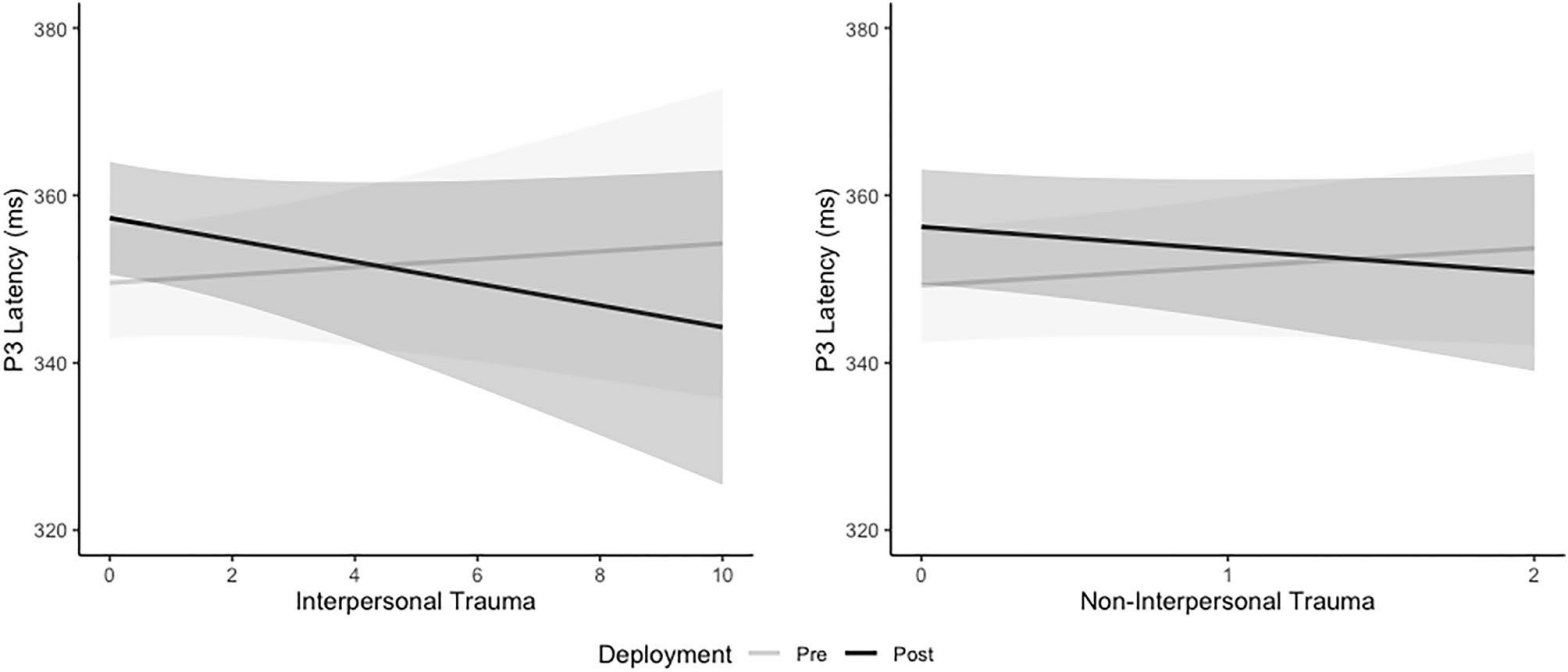
Estimated Marginal Trends (with 95% Confidence Intervals) from the linear mixed model for P3 Latency by Interpersonal and Non-interpersonal Cumulative Trauma Type across Deployment.

The Deployment*Interpersonal CTT interaction (Fig. 5) shows that after accounting for other variables in the linear mixed model, P3 latency increased with increasing Interpersonal CTT at pre-deployment compared to decreasing at post-deployment and the effect was moderate (b = 1.78, *p* = 0.015, Cohen’s d = 0.43). P3 latency at pre-deployment increased by 0.47ms, 95%CI (− 1.56, 0.2.50) with every 1 additional Interpersonal CTT whereas it decreased by 1.31ms, 95%CI (− 0.3.38, 0.77) at post-deployment. Similarly, the Deployment*Non-interpersonal CTT interaction (Fig. 5) shows P3 latency increased with increasing Non-interpersonal CTT at pre-deployment compared to decreasing at post-deployment and the effect was large (b = 4.95, *p* = 0.013, Cohen’s d = 1.19). P3 latency at pre-deployment increased by 2.23ms, 95%CI (− 3.1, 7.55) with every 1 trauma increase in Non-interpersonal CTT compared to a decrease at post-deployment of 2.72, 95%CI (− 8.15, 2.71).

**Figure 6:**
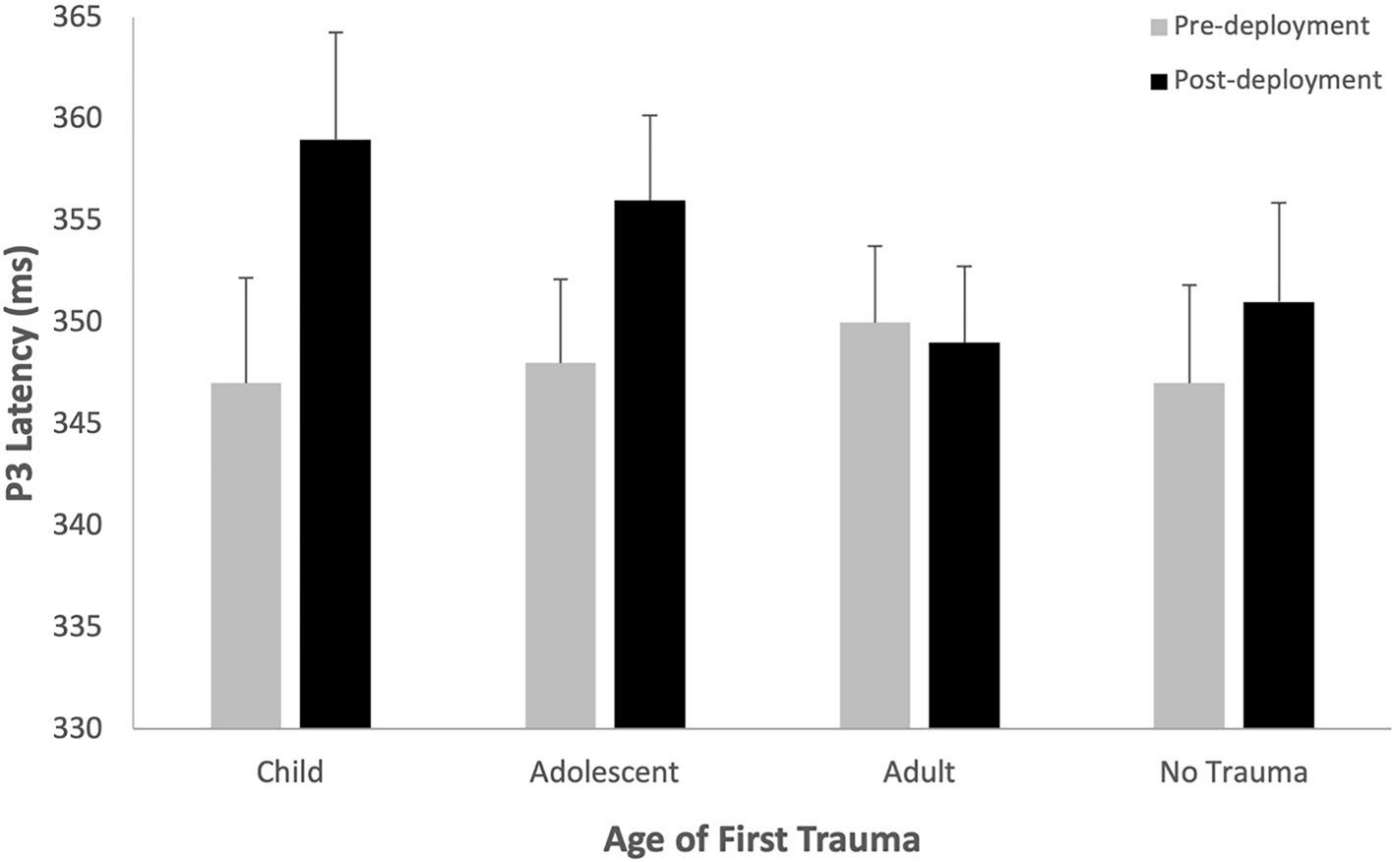
Estimated Marginal Means (with Standard Error) from the linear mixed model for P3 Latency by Trauma-Onset across Deployment.

The Deployment*Trauma-onset interaction (Fig. 6) shows that after accounting for other variables in the linear mixed model, there was a significantly longer P3 latency for the childhood-onset (b = 12.17, SE = 3.54, *p* < 0.001) and adolescent-onset (b = 7.90, SE = 2.36, *p* < 0.001) groups across deployment and change in P3 latency was significant greater than the adult-onset trauma group (Childhood: b = 12.91, SE = 4.04, *p* = 0.002; Adolescent: b = 8.64, SE = 3.08, *p* = 0.005). There was no difference in P3 latency between trauma groups at pre- or post-deployment (refer to Supplementary Materials).

### Post traumatic stress disorder symptoms (PCL) and sub-cluster correlations

Table 5 outlines correlations between PCL and sub-clusters with ERP component and cumulative trauma load at pre- and post-deployment. At pre-deployment, smaller N2 amplitude was associated with higher arousal symptoms and higher interpersonal CTT was associated with higher overall PCL and higher re-experiencing symptoms.

**Table 5:** Spearman’s rho correlation coefficients for PCL (and subclusters) by ERP, and Cumulative Trauma Type at pre- and post-deployment.

	Overall	Re-experiencing	Avoidance	Arousal
Pre-deployment				
N2 amplitude	0.13	0.09	0.60	0.18*
N2 latency	0.08	0.00	0.03	0.06
P3 amplitude	0.10	0.05	0.07	0.13
P3 latency	− 0.02	0.02	− 0.02	− 0.07
Interpersonal CTT	0.26*	0.30***	0.22	0.20
Non-interpersonal CTT	0.12	0.19	0.06	0.07
Post-deployment				
N2 amplitude	0.10	0.13	0.09	0.06
N2 latency	0.00	0.02	0.06	− 0.04
P3 amplitude	0.00	0.08	− 0.06	0.02
P3 latency	− 0.04	− 0.07	− 0.06	0.01
Interpersonal CTT	0.20	0.13	0.11	0.18
Non-interpersonal CTT	0.17	0.08	0.11	0.17

*CTT* Cumulative Trauma Types, N2 is negative ERP component so smaller magnitude is associated with increasing N2 amplitude. P-values are Bonferroni adjusted.

***p < 0.001; **p < 0.01; *p < 0.05.

## Discussion

This paper was the first to our knowledge to examine the impact of pre-deployment timing and cumulative type of trauma and posttraumatic stress disorder (PTSD) symptoms on inhibitory control processing across military deployment. In line with our hypotheses, (1) timing of trauma explained additional variance over and above cumulative trauma but only for response inhibition, (2) high interpersonal trauma load was associated with enhanced conflict monitoring across deployment, and (3) developmental (child/adolescent) trauma impaired response suppression across deployment. Contrary to our hypotheses, timing of trauma (including developmental trauma) did not impact on conflict monitoring and higher interpersonal trauma load showed a faster response inhibition. These findings are explored below.

In line with the cumulative stress model^7,8^, this study found cumulative trauma load affected the N2 and P3 component suggesting an impact on conflict monitoring (detecting and controlling conflict between incoming stimuli) and response suppression (inhibiting an activated response). Furthermore, higher trauma load, particularly interpersonal, was associated with childhood trauma onset and higher PTSD symptoms, especially re-experiencing symptoms. Although developmental trauma was not a significant predictor of conflict monitoring as hypothesized these results are consistent with previous research showing exposure to multiple types of trauma, particularly interpersonal, is associated with early-onset trauma, PTSD and impaired inhibitory control^9–11,13–15^.

More specifically, we found those with higher interpersonal trauma displayed reduced resources toward conflict monitoring (smaller N2 amplitude) at pre-deployment, which was associated with arousal symptoms. This suggests high interpersonal trauma led to less resources for monitoring competing task-relevant stimuli, potentially due to difficulty allocating resources away from arousal symptoms towards processing the task at hand^4^. In contrast, when considering changes across deployment, this group increased resources toward monitoring conflict (increased N2 amplitude) and reduced resources toward response suppression (smaller P3 amplitude and faster P3 latency). Increased resources for conflict monitoring has previously been found in veterans with PTSD and is consistently associated with anterior cingulate cortex (ACC) hyperactivation^19,23–25^. The ACC is activated when anticipating traumatic stimuli in PTSD^42,43^, suggesting high interpersonal trauma may create difficulty regulating threat processing and reactivity or increases intrusive thoughts during deployment which impairs inhibitory control and creates a vulnerability to PTSD. Reduced resources toward response suppression following high interpersonal trauma supports literature showing smaller NoGo-P3 amplitude and hypoactivation in the ACC and orbito-medial prefrontal cortex to the NoGo-P3 is linked with early-onset trauma, PTSD and difficulties in cognitive control and decision making^6,29,38–41^. Taken together, high interpersonal trauma may lead to an overwhelmed cognitive system that results in increased effort for monitoring and detecting conflict between activated stimuli in their environment (hypervigilance toward threat) during deployment, thus depleting resources for inhibiting responses. This is consistent with evidence showing PTSD in a military population is associated with difficulty disengaging from internal and external distractions and inhibiting automatic responses^5^.

Contrary to interpersonal trauma, higher non-interpersonal trauma load was associated with delayed speed of conflict monitoring at pre-deployment, and this was not associated with PTSD symptoms. This provides further support for interpersonal trauma being more predictive of PTSD than non-interpersonal trauma^9–13^. Furthermore, in our study, significant changes associated with non-interpersonal trauma in relation to allocation of resources toward conflict monitoring and response suppression were seen in those without non-interpersonal trauma rather than high non-interpersonal trauma. This suggests non-interpersonal trauma has little impact on inhibitory control in the early aftermath of deployment.

Consistent with our hypotheses, trauma onset in adolescence resulted in increased resources toward response suppression and delayed response suppression processing across deployment (increased No-Go-P3 amplitude and longer NoGo-P3 latency). This supports previous research in police officers with sub-clinical PTSD and veterans with PTSD respectively^26,27^. Similar to adolescent trauma, we found childhood trauma resulted in a delayed, but not enhanced, response suppression across deployment. Longer latency for response suppression following childhood and adolescent trauma suggests the need for more time during response suppression processing in order to inhibit the correct response. Although we found no association between delayed response suppression and PTSD in the early aftermath of deployment (4 months), delayed response inhibition has been associated with PTSD, particularly arousal and re-experiencing symptoms, in the years following deployment^27^. This suggests those with trauma onset in childhood and adolescence may be more vulnerable to developing PTSD in the long-term aftermath of deployment.

The NoGo-P3 activates pre-frontal regions, including the orbitofrontal cortex, which develop during adolescence, alongside developing connectivity between frontal inhibitory and the amygdala and threat detection networks^19,23,24,30^. As the amygdala and threat detection networks develop in childhood, this may suggest childhood trauma impairs speed of response suppression due to increased arousal symptoms from an impaired emotion regulation system, and adolescent trauma impairs allocation of resources toward response suppression and speed of response suppression through impaired frontal inhibitory connectivity during adolescence^30–35^. Further research is needed to determine the brain networks and connectivity contributing to impaired response suppression following adolescence and childhood trauma.

Taken together, these findings indicate that like brain injury during development^36^, developmental trauma also has a long-term consequence on inhibitory control. Our findings support the sensitive period of trauma exposure model^16,17^, where developmental trauma appears to impact on inhibitory frontal networks or connections, which are important for response suppression^14,33,34^ and is consistent with research showing developmental trauma leads to decreased accuracy for inhibiting responses and increased impulsivity and risk-taking behavior^30,44,45^. Furthermore, it suggests developmental trauma plays a differential and supplementary role to cumulative trauma load during inhibitory control processing.

By adulthood, brain development is largely complete, and we found trauma onset in adulthood did not appear to impact on inhibitory control processing in the early aftermath of deployment. However, adults without trauma exposure displayed decreased resource allocation for response suppression across deployment, but unlike those with high interpersonal trauma they did not get faster at response suppression. The percentage of high PTSD symptoms in those without trauma exposure was 13% at post-deployment and this group went from reporting the lowest to highest avoidance across deployment. Increased PTSD symptoms have been associated with increased attentional threat avoidance during acute stress on deployment^46,47^. Therefore, lack of trauma exposure and low deployment experience may increase avoidance to threat leading to reduced inhibitory suppression processes and a temporary increase in PTSD symptoms in the early aftermath of post-deployment.

Although our findings support and extend current research, there are several limitations. Larger sample size would provide greater power to further differentiate interpersonal trauma types and differentiate individuals with childhood and adolescent onset trauma from childhood onset trauma alone^9,14^. Further, our models explain up to 10% of variance in the ERP components suggesting scope for a wider range of predictors to further understand mechanisms contributing to inhibitory control.

Our research highlights the need for the development and implementation of tailored strategies for strengthening emotion regulation, inhibitory control, and prefrontal functioning in military personnel, particularly those with (a) developmental trauma, (b) high interpersonal trauma load and (c) no prior trauma exposure who display impaired inhibitory control across deployment and may be at risk for PTSD. High post-deployment PTSD symptoms and combat exposure were not significant predictors of conflict monitoring or response suppression in our study, suggesting they did not impact on inhibitory control processing in the immediate post-deployment period. As PTSD can change following deployment, or be delayed, our research highlights the importance of further follow-up at least one-year post-deployment to explore relationships between timing of trauma and trauma load on inhibitory control and PTSD trajectories, consistent with previous research^48–51^.

In conclusion, this study investigated the impact of timing and cumulative type of trauma as well as PTSD symptoms on inhibitory control processing across military deployment. Our findings extend previous research by showing the supplementary and differential role of interpersonal trauma load alongside timing of trauma on inhibitory control processing. Where developmental trauma appears to impact on response suppression, interpersonal trauma leads to an overwhelmed inhibitory system that impairs conflict monitoring and response suppression. Our findings also reveal a differential impact of childhood and adolescent trauma on response suppression, which highlights the need for research to examine specific critical periods rather than defining trauma before age 18 as childhood trauma. Taken together, this paper supports a combined cumulative trauma and sensitive period of trauma exposure model for inhibitory control processing and highlights the enduring impact of timing of trauma and trauma load on inhibitory control.

## Methods

### Participants

Participants were 166 predominately male Australian Defence Force army combat personnel (mean age = 28, SD = 7) deployed to the Middle East Area Operations (MEAO) between 2010 and 2012 and recruited for the MEAO Prospective Study led by the Centre for Military and Veterans’ Health, and the Department of Defence and Department of Veterans’ Affairs. The MEAO Prospective Study is outlined in full detail in Davy, Dobson^52^. Psychological and neurocognitive assessments were completed approximately 3-months prior to, and 4-months after return from, deployment.

### Ethics

Written informed consent was obtained from all participants. The study received approval by the Australian Defence Human Research Ethics Committee (Protocol no. 488-07), and University of Adelaide Human Research Ethics Committee (Protocol no. H-064-2008). The current study is a secondary analysis and is not part of the original MEAO Prospective Study protocol. The study was carried out in accordance with relevant guidelines and regulations.

### Measures

Demographic data was collected at pre-deployment including age, binary sex (male, female), and number of prior deployments. Self-reported Mild Traumatic Brain Injury (mTBI) was also collected at pre- and post-deployment. For analysis, the presence of mTBI at pre and/or post-deployment was added as a covariate, along with age at pre-deployment and sex.

PTSD symptoms were assessed at pre- and post-deployment using the Posttraumatic Stress Disorder Checklist—Civilian (17-item; PCL-C)^53^, based on DSM-IV PTSD criteria with re-experiencing, avoidance, and arousal sub-clusters. At the time of the MEAO study, the PCL-C was the gold standard in assessing PTSD with good reliability and validity in veterans and cut-off values above 30 indicating possible PTSD^54^. Because of the low incidence of PTSD in this sample, individuals were split into low PTSD symptoms (PCL < 30) and high PTSD symptoms (PCL $\ge 30$) for analysis.

Pre-deployment trauma exposure was measured across 18 trauma types, including direct combat, accident/unexpected traumas, sexual traumas, and other interpersonal traumas, adapted for the MEAO study (refer to Supplementary Materials). Scale items were derived from the Composite International Diagnostic Interview v2.1 trauma module^55^, and previous community studies^56^. Responses were summed to create cumulative trauma type (range 0–18) and split into two variables: the sum of interpersonal cumulative trauma type, and the sum of non-interpersonal cumulative trauma type. Age of onset and offset for each trauma type was recorded. Trauma onset was set at first onset of any trauma and grouped into four categories; childhood-onset (before 10 years); adolescent-onset (10–17 years), adult-onset (18 years onwards) or no pre-deployment trauma.

Post-deployment combat exposure was measured for specific experiences, including participation in armed combat or combat environments, proximity to serious injury or death, and exposures evoking emotion (refer to Supplementary Materials)^57^. Scale items were derived from the Australian Gulf War Veterans’ Health Study^58^, Deployment Risk and Resilience Inventory-2 (DRRI-2; Ref.^59^), and Traumatic Stressors Exposure Scale (TSES; Ref.^60^). Combat exposure along a 5-point scale (0 = never, 4 = 10 + experiences) was summed across 26 items (range 0–104), with higher scores indicating greater combat exposure on deployment.

### Go/NoGo paradigm

EEG data from the Go/NoGo paradigm was collected by LabNeuroTM platform (Brain Resource Ltd., Sydney, Australia) at pre- and post-deployment^52^. In this 5-min paradigm, participants were shown the word ‘PRESS’ repeatedly for 500ms and instructed to withhold response for words appearing in red, or respond manually (using the index finger of each hand) for words appearing in green, as fast and accurately as possible. The word was presented six times in a row in the same color along with 28 pseudorandom sequences (21 green and 7 red).

### EEG data collection and analysis

EEG data was recorded using Quik-Cap and 40 channel NuAmps with Ag/AgCl sintered electrodes located according to the International 10–20 system from the 26 central scalp sites, and 500Hz sampling rate. Data were collected from four electro-oculogram (EOG) channels for detection of eye movement artefacts with correction undertaken offline according to Gratton, Coles^61^. This procedure estimates correction factors from EOG and EEG records during the experimental session to estimate a propagation factor to present the relationship between EOG and EEG traces and computes separate correction factors for blinks and eye movements^61^.

Average ERPs were calculated for the NoGo Trial. Individual single-trial ERP epochs were filtered with low-pass Tukey (cosine) filter (− 0.01–25 Hz) that attenuated frequencies above 25Hz. Single trials were averaged, and peak components identified within defined latency windows according to previous analyses using this dataset^62^ and validated by visual inspection across individual participants at each site. Baseline to peak method was used to score ERP components. The NoGo-N2 (maximum negative peak 180-220ms post-stimulus) was analyzed at frontocentral sites (FCz and Cz), and NoGo-P3 (maximum positive peak 230-450ms) at midline sites (Cz, Fz and Pz).

### Statistical analysis

We used Linear Mixed Modelling as it has advantages over repeated measures ANOVA when modelling complex data, with missingness and an unbalanced and/or nested structure, and it allows for consideration of multiple random variables (i.e., participant and topographic site)^63,64^. Mixed models were fit using the *lmer* function from the *lme4* package in R version 3.6.0^65^ using the Kenward-Roger’s method and p-values from post-hoc tests were adjusted using the Bonferroni method. Restricted maximum likelihood (REML) was chosen over maximum likelihood (ML) as it maximises the variance rather than the mean parameter and is preferred for smaller sample sizes and complex mixed models^66^.

Amplitude and latency were fit separately for the N2 and P3 components with a random intercept for Subject with Topographic Site (1|StudyID/Site). Sex, mTBI, age, and combat exposure were selected as covariates in the baseline model. Our baseline model for each ERP was:

$$ERP \sim mTBI + Sex +\mathrm{ Age}+ Combat \,Exposure +\mathrm{ Site}+ Deployment + (1|StudyID/Site)$$

An additive approach was taken to model building. From the baseline model we firstly added the interaction of Deployment*Cumulative trauma type (split into summed variables of interpersonal cumulative trauma, and non-interpersonal cumulative trauma), followed by Deployment*Trauma-onset (Trauma < 10 years, Trauma 10–17, Trauma 18 + , No Trauma), to determine the additive improvement in model fit of cumulative trauma type and trauma-onset on the response variable. Lastly, we added a 3-way interaction of Deployment and PTSD symptoms (Low, High) with cumulative trauma and trauma onset. For model fit comparison, the *KRmodcomp* function, an approximate F-test based on the Kenward-Roger approach^67^ was used from the *pbkrtest* package in R^68^.

Differences between trauma groups were compared for demographic data using ANOVA for continuous response variables and Chi-square test using the fisher method and simulated p-values due to low cell counts for categorical response variables. The *cor.test* function from the *stats* package in R version 3.6.0^65^ was used to test the correlation of PCL (and sub-clusters) with ERP amplitude and latency, and cumulative trauma type. The Spearman’s (rho) method was used due to non-normality of PCL and cumulative trauma type and p-values were adjusted using the Bonferroni correction.

### Supplementary Information

Supplementary Tables.

## Data Availability

The data that support the findings of this study are available from the Australian Department of Defence and Joint Health Command but restrictions apply to the availability of these data, which were used under license for the current study, and so are not publicly available. Data are however available from the author, L.M, upon reasonable request and with permission of the Australian Department of Defence and Joint Health Command.
