# Supplementary material for: Cumulative trauma load and timing of trauma prior to military deployment differentially influences inhibitory control processing across deployment
Source: Sci Rep. 2023 Dec 5;13:21414. doi: 10.1038/s41598-023-48505-7 (PMC10696090; doi:10.1038/s41598-023-48505-7)
Supplement: Supplementary file 1 — Supplementary Tables. [file 41598_2023_48505_MOESM1_ESM.pdf]

SUPPLEMENTARY MATERIALS

1 DEMOGRAPHICS

Table A1. Adapted Questionnaire assessing Trauma Exposures in MEAO Study

Please indicate if you have experienced any of the following events:

**Event**
Direct combat
Life-threatening accident
Fire, flood, or other natural disaster
Witnessed someone badly injured or killed
Rape
Sexual Molestation
Serious physical attack or assault
Threatened / harassed without weapon
Threatened with weapon / held captive / kidnapped
Tortured or victim of terrorists
Domestic violence
Witnessed domestic violence
Finding a dead body
Witnessed someone suicide or attempt suicide
Child abuse - physical
Child abuse - emotional
Any other stressful event, please specify:
Did you ever suffer a great shock because any of these events happened to someone close to you? Please specify event type.

Response for each event:

- Experienced Event: Yes, No
- Number of Times
- Age first time
- Age last time

Table A2. Adapted Questionnaire assessing Combat Exposure on deployment from the MEAO Study

During your last deployment to the MEAO, how often …?

1	Did you clear/ search buildings?
2	Did you clear / search caves?
3	Did you come under small arms or anti-aircraft fire?
4	Did you come under guided or directed mortar/artillery fire or missile attack?
5	Did you experience in-direct fire (e.g. rocket attack)?
6	Did you seriously fear you would encounter an IED?
7	Did you experience an IED/EOD that detonated?
8	Did you experience a suicide bombing?
9	Did you experience a landmine strike?
10	Did you encounter small arms fire from an unknown enemy combatant (e.g. sniper, civilian with weapon)?
11	Did you discharge your weapon in direct combat?
12	Did you experience a threatening situation where you were unable to respond due to the rules of engagement?
13	Did you go on combat patrols or missions?
14	Did you participate in support convoys (e.g. re-supply, VIP escort)?
15	Were you concerned about yourself or others (including allies) having an unauthorized discharge of a weapon?
16	Were you in danger of being killed? E.g. combat, motor vehicle accident (MVA), assault, hostage situation
17	Were you in danger of being injured? E.g. combat, MVA, assault, hostage situation
18	Did you handle dead bodies? E.g. combat civilian casualties
19	Did you see dead bodies? E.g. combat civilian casualties.
20	Did you hear of a close friend or co-worker who had been injured or killed. E.g. combat, MVA, disaster situation
21	Were you present when a close friend or co-worker was injured or killed. E.g. combat, MVA, disaster situation
22	Were you witness to human degradation and misery on a large scale? E.g. refugee camps, starvation
23	Did you hear of a loved one who had been injured or killed?
24	Were you present when a loved one was injured or killed?
25	Do you believe your action or inaction resulted in someone being seriously injured? E.g. in combat or as a result of rules of engagement or UN restrictions not allowing you to act
26	Did you believe your actions or inactions resulted in someone being killed? E.g. in combat or as a result of rules of engagement or UN restrictions not allowing you to act

Response options for Questions 1-25: Never, Once, 2-4 times, 5-9 times, 10+ times.

Table A3: Comparison of demographic data by Trauma Onset

	Childhood Trauma	Adolescent Trauma	Adult Trauma	No Prior Trauma	F value	DF	p
N	20	45	57	44			
Pre-Deployment							
Male	95%	98%	96%	95%			.891
Age	28.5 (6.32)	28.24 (6.44)	31.72 (7.16)	25.49 (6.45)	7.08	3	<.001
Ever Deployed	65%	76%	86%	34%			<.001
Times Deployed	2.56 (2.64)	2.26 (2.46)	4.96 (4.42)	0.79 (1.08)	12.98	3	<.001
Trauma Types	4.75 (2.69)	3.56 (1.99)	3.02 (2.07)	0 (0)	4.80	2	.010
Interpersonal	4.00 (2.64)	2.64 (1.82)	2.44 (1.91)	0 (0)	4.59	2	.012
Non-Interpersonal	0.75 (0.72)	0.91 (0.70)	0.58 (0.68)	0 (0)	2.89	2	.059
PCL	20.4 (5.46)	20.4 (5.40)	19.96 (5.76)	18.03 (2.38)	1.93	3	.126
Re-experiencing	5.8 (1.88)	5.73 (1.21)	5.58 (1.39)	5.15 (0.81)	1.76	3	.156
Avoidance	8.7 (2.52)	8.24 (2.77)	8.19 (2.66)	7.31 (0.83)	1.95	3	.124
Arousal	5.9 (1.52)	6.42 (2.17)	6.19 (2.3)	5.56 (1.23)	1.47	3	.225
Post-Deployment							
PCL	21.35 (4.3)	24.63 (9.28)	22.1 (7.68)	23.44 (12.12)	0.78	3	.508
Re-experiencing	6.06 (1.34)	6.80 (2.53)	6.23 (1.74)	6.77 (3.8)	0.65	3	.587
Avoidance	8.29 (2.17)	9.41 (3.37)	8.81 (3.59)	9.54 (5.26)	0.57	3	.636
Arousal	7.00 (1.87)	8.41 (3.93)	7.06 (3.00)	7.13 (3.59)	1.57	3	.201
Combat Exposure	30.59 (16.74)	34.9 (15.91)	43.47 (15.67)	27.95 (15.98)	7.37	3	<.001
High PTSDS	6%	24%	8%	13%			.135
mTBI	44%	47%	44%	22%			.102

Note: PCL: Post-traumatic stress symptoms; mTBI: Mild Traumatic Brain Injury
All values refer to mean (standard deviation), unless indicated otherwise as percentage.
P-values from ANOVA for continuous variables and Fisher's test for categorical variables.

Table A4: Post-hoc paired comparison test by trauma onset for significant overall comparisons from Table SM1

Group1	Group2	Age	Time Deployed	Combat Exposure	All Trauma Types	Interpersonal Trauma Types	Ever Deployed
Child	Adolescent	.887	.741	.352	.042	.014	.504
Child	Adult	.066	.006	.005	.003	.004	.023
Adolescent	Adult	.010	.000	.014	.214	.609	.058
No Trauma	Child	.101	.058	.570			.084
No Trauma	Adolescent	.058	.046	.055			.002
No Trauma	Adult	<.001	<.001	<.001			<.001

Note: All values are paired p-values from paired t-test for continuous variables and paired fisher's test for categorical variable (ever deployed).

2 MIXED LINEAR MODELLING ADDITIONAL ANALYSES

Table B1: Estimated Marginal Means for significant main effect and interactions from the linear mixed model for N2 Amplitude.

	Estimate	SE	df	95% Lower CI	95% Upper CI
Main Effects					
Site					
Cz	0.63	0.89	134	-1.39	2.64
FCz	-0.16	0.89	134	-2.17	1.85
Deployment					
Pre-deployment	0.95	0.887	133	-1.06	2.96
Post-deployment	-0.483	0.889	134	-2.5	1.53
CTT_InT	0.19	0.19	131	-0.19	0.57
CTT_NInT	-0.51	0.53	131	-1.55	0.54
2-way Interaction					
CTT_InT					
Pre-deployment	0.34	0.20	157	-0.12	0.79
Post-deployment	0.04	0.20	162	-0.42	0.50
CTT_NInT					
Pre-deployment	-0.99	0.56	160	-2.24	0.27
Post-deployment	-0.03	0.56	165	-1.30	1.24

Note: CTT_InT: Cumulative Interpersonal Trauma Type
CTT_NInT: Cumulative Non-Interpersonal Trauma Type

Table B2: Contrasts for significant main effect and interactions from the linear mixed model for N2 Amplitude.

	Estimate	SE	df	t	p	Cohens d
Main Effects						
Site						
Cz - FCz	0.79	0.24	386	3.34	0.001	0.17
Deployment						
Pre-post deployment	1.43	0.24	390	6.01	<.0001	0.53
2-way Interaction						
CTT_InT						
Pre-Post deployment	0.30	0.12	394	2.39	0.018	0.06
CTT_NInT						
Pre-Post deployment	-0.96	0.36	391	-2.64	0.009	-0.20

Note: p-values are Bonferroni adjusted
CTT_InT: Cumulative Interpersonal Trauma Type
CTT_NInT: Cumulative Non-Interpersonal Trauma Type

Table B3: Estimated Marginal Means for significant main effect and interactions from the linear mixed model for N2 Latency

	Estimate	SE	df	95% Lower CI	95% Upper CI
Main Effects					
Deployment					
Pre-deployment	281	4.35	134	271	291
Post-deployment	273	4.36	136	263	283
Age	0.82	0.26	131	0.30	1.34
CTT_NInT.	3.83	2.58	131	-1.28	8.95
2-way Interactions					
CTT_NInT					
Pre-deployment	7.55	2.76	170	1.30	13.79
Post-deployment	0.12	2.79	176	-6.19	6.40

Note: p-values are Bonferroni adjusted
CTT_NInT: Cumulative Non-Interpersonal Trauma Type

Table B4: Contrasts for significant main effect and interactions from the linear mixed model for N2 Latency

	Estimate	SE	df	t	p	Cohens d
Main Effects						
Deployment						
Pre-Post-deployment	7.59	1.34	392	5.67	<.001	0.32
2-way Interactions						
CTT_NInT						
Pre-Post-deployment	7.42	2.03	392	3.65	<.001	0.31

Note: p-values are Bonferroni adjusted
CTT_NInT: Cumulative Non-Interpersonal Trauma Type

Table B5: Estimated Marginal Means for significant main effect and interactions from the linear mixed model for P3 Amplitude

	Estimate	SE	df	95% Lower CI	95% Upper CI
Main Effects					
Site					
Cz	7.84	0.75	135	6.02	9.66
Fz	6.33	0.75	135	4.51	8.16
Pz	6.95	0.75	135	5.13	8.78
Deployment					
Pre-deployment	7.12	0.75	131	5.43	8.81
Post-deployment	6.96	0.75	132	5.27	8.66
Trauma Onset					
Childhood	8.09	1.11	127	5.89	10.30
Adolescent	7.58	0.90	127	5.80	9.36
Adult	6.52	0.82	127	4.91	8.14
No Trauma	5.97	1.05	127	3.89	8.05
CTT_InT	-0.22	0.19	128	-0.59	0.15
CTT_NInT	-1.05	0.49	128	-2.02	-0.08
2-way Interactions					
Pre-Deployment					
Childhood	8.16	1.15	144	5.89	10.43
Adolescent	7.09	0.92	138	5.28	8.91
Adult	6.57	0.83	136	4.92	8.22
No Trauma	6.65	1.08	140	4.52	8.79
Post Deployment					
Childhood	8.02	1.16	147	5.74	10.30
Adolescent	8.07	0.92	141	6.24	9.89
Adult	6.48	0.84	139	4.83	8.13
No Trauma	5.29	1.08	142	3.15	7.43
CTT_InT					
Pre-deployment	-0.05	0.20	152	-0.50	0.39
Post-deployment	-0.39	0.20	159	-0.84	0.06
CTT_NInT					
Pre-deployment	-1.39	0.51	155	-2.55	-0.23
Post-deployment	-0.70	0.52	160	-1.88	0.47

Note: CTT_InT: Cumulative Interpersonal Trauma Type
CTT_NInT: Cumulative Non-Interpersonal Trauma Type

Table B6: Contrasts for significant main effect and interactions from the linear mixed model for P3 Amplitude

		Estimate	SE	df	t	p	Cohens d
Main Effect							
Site	Cz-Fz	1.51	0.24	268	6.36	<.001	0.36
	Cz-Pz	0.89	0.24	269	3.75	0.001	0.21
	Fz-Pz	-0.62	0.24	270	-2.61	0.029	-0.15
Deployment	Pre-Post	0.16	0.21	387	0.76	0.450	0.04
Trauma Onset	Childhood - Adolescent	1.12	0.66	386	1.68	0.093	0.12
	Childhood - Adult	0.05	0.67	387	0.08	0.940	0.38
	Adolescent - Adult	-1.06	0.51	391	-2.09	0.037	0.25
	No Trauma - Childhood	1.23	0.84	387	1.45	0.147	-0.51
	No Trauma - Adolescent	2.34	0.70	389	3.36	0.001	-0.39
	No Trauma - Adulthood	1.28	0.60	389	2.12	0.034	-0.13
2-way Interactions							
Deployment							
Pre	Childhood - Adolescent	1.07	1.05	154	1.02	0.741	0.26
	Childhood - Adult	1.59	1.12	150	1.42	0.490	0.38
	Adolescent - Adult	0.52	0.84	152	0.63	0.923	0.13
	No Trauma - Childhood	-1.51	1.35	154	-1.12	0.678	-0.36
	No Trauma- Adolescent	-0.44	1.11	154	-0.40	0.979	-0.11
	No Trauma- Adult	0.08	1.02	151	0.08	1.000	0.02
Post	Childhood - Adolescent	-0.05	1.06	158	-0.04	1.000	-0.01
	Childhood- Adult	1.54	1.14	156	1.36	0.527	0.37
	Adolescent- Adult	1.59	0.85	159	1.88	0.242	0.38
	No Trauma - Childhood	-2.74	1.36	157	-2.02	0.187	-0.66
	No Trauma - Adolescent	-2.78	1.12	159	-2.49	0.066	-0.67
	No Trauma - Adult	-1.19	1.02	155	-1.17	0.650	-0.29
Pre-post	CTT_InT	0.34	0.12	391	2.82	0.005	0.08
	CTT_NInT	-0.69	0.33	389	-2.11	0.036	-0.17
	Childhood	0.14	0.59	384	0.24	0.809	0.03
	Adolescent	-0.97	0.39	390	-2.49	0.013	-0.23
	Adult	0.09	0.33	391	0.28	0.784	0.02
	No Trauma	1.37	0.49	390	2.79	0.006	0.33
	Childhood - Adolescent	1.12	0.66	386	1.68	0.093	
	Childhood - Adult	0.05	0.67	387	0.08	0.940	
	Adolescent - Adult	-1.06	0.51	391	-2.09	0.037	
	No Trauma - Childhood	1.23	0.84	387	1.45	0.147	
	No Trauma - Adolescent	2.34	0.70	389	3.36	0.001	
	No Trauma - Adult	1.28	0.60	389	2.12	0.034	

Note: p-values are Bonferroni adjusted
CTT_InT: Cumulative Interpersonal Trauma Type
CTT_NInT: Cumulative Non-Interpersonal Trauma Type

Table B7: Estimated Marginal Means from the linear mixed model for P3 Latency for significant main effect and interactions.

	Estimate	SE	df	95% Lower CI	95% Upper CI
Main Effects					
Site					
Cz	352	3.36	142	346	359
Fz	354	3.36	142	347	360
Pz	347	3.37	143	340	354
Deployment					
Pre-deployment	348	3.32	135	341	355
Post-deployment	354	3.33	136	347	360
Trauma Onset					
Childhood	353	4.92	127	343	363
Adolescent	352	3.97	127	344	360
Adult	349	3.62	127	342	356
No Trauma	349	4.64	126	340	358
CTT_InT	-0.42	0.83	128	-2.07	1.23
CTT_NInT	-0.25	2.16	128	-4.53	4.03
2-way Interaction					
Pre-Deployment					
Childhood	347	5.2	158	337	357
Adolescent	348	4.12	147	340	357
Adult	350	3.74	146	342	357
No Trauma	347	4.85	151	338	357
Post-Deployment					
Childhood	359	5.26	164	349	369
Adolescent	356	4.17	153	348	365
Adult	349	3.77	149	341	356
No Trauma	351	4.88	154	342	361
CTT_InT					
Pre-deployment	0.47	0.899	174	-1.56	2.504
Post-deployment	-1.31	0.918	185	-3.38	0.768
CTT_NInT					
Pre-deployment	2.23	2.35	179	-3.1	7.55
Post-deployment	-2.72	2.4	191	-8.15	2.71

Note: CTT_InT: Cumulative Interpersonal Trauma Type
CTT_NInT: Cumulative Non-Interpersonal Trauma Type

Table B8: Contrasts from the linear mixed model for P3 Latency for significant main effect and interactions.

		Estimate	SE	df	t	p	Cohens d
Main Effect							
Site	Cz-Fz	-1.36	1.40	643	-0.97	1.00	-0.33
	Cz-Pz	5.24	1.41	644	3.71	0.00	1.26
	Fz-Pz	6.60	1.42	645	4.65	<.001	1.59
Deployment	Pre-Post						
Trauma Onset	Childhood - Adolescent	-3.03	4.66	127	-0.65	1.00	-0.15
	Childhood - Adult	0.11	4.30	128	0.03	1.00	0.01
	Adolescent - Adult	0.53	4.44	127	0.12	1.00	0.03
	No Trauma - Childhood	-5.90	1.25	651	-4.71	<.001	-0.28
	No Trauma - Adolescent						
	No Trauma - Adulthood	-3.57	5.67	126	-0.63	1.00	-0.17
2-way Interactions							
Deployment							
Pre	Childhood - Adolescent	-1.60	4.83	177	-0.33	0.987	-0.08
	Childhood - Adult	-2.78	5.13	171	-0.54	0.949	-0.13
	Adolescent - Adult	-1.18	3.82	174	-0.31	0.990	-0.06
	No Trauma - Childhood	0.39	6.18	177	0.06	1.000	0.02
	No T- Adolescent	-1.21	5.07	177	-0.24	0.995	-0.06
	No T- Adult	-2.39	4.64	172	-0.52	0.956	-0.11
Post	Childhood - Adolescent	2.61	4.70	178	0.56	0.945	0.13
	Childhood- Adult	2.67	4.91	186	0.54	0.948	0.13
	Adolescent- Adult	10.13	5.24	183	1.93	0.218	0.49
	No Trauma - Childhood						
	No Trauma - Adolescent	-7.52	6.25	184	-1.20	0.626	-0.36
	No Trauma - Adult	-4.86	5.16	186	-0.94	0.782	-0.23
Pre-post	CTT_InT	4.95	1.99	652	2.49	0.013	1.19
	CTT_NInT	1.78	0.73	652	2.45	0.015	0.43
	Childhood	-12.17	3.54	649	-3.43	0.00	-0.58
	Adolescent	-7.90	2.36	654	-3.35	0.00	-0.38
	Adult	0.74	2.02	655	0.37	0.72	0.04
	No Trauma	-4.26	2.94	648	-1.45	0.15	-0.20
	Childhood - Adolescent	-4.27	4.00	650	-1.07	0.286	
	Childhood - Adult	-12.91	4.04	652	-3.19	0.002	
	Adolescent - Adult	-8.64	3.08	654	-2.80	0.005	
	No Trauma - Childhood	7.91	5.08	647	1.56	0.120	
	No Trauma - Adolescent	3.64	4.19	651	0.87	0.385	
	No Trauma - Adult	-5.00	3.63	650	-1.38	0.169	

Note: p-values are Bonferroni adjusted.
CTT_InT: Cumulative Interpersonal Trauma Type
CTT_NInT: Cumulative Non-Interpersonal Trauma Type

Table B9. Spearman's Correlation for PCL (and sub-clusters) at pre-deployment by ERP Component and Cumulative Trauma.

PCL	Variable	ρ	p-value	adjusted p-value
Overall	N2 Amplitude	0.13	0.022	0.528
	N2 Latency	0.08	0.181	1.000
	P3 Amplitude	0.10	0.025	0.600
	P3 Latency	-0.02	0.634	1.000
	CTT_InT	0.26	0.001	0.024
	CTT_NInT	0.12	0.140	1.000
Re-experiencing	N2 Amplitude	0.09	0.112	1.000
	N2 Latency	0.00	0.985	1.000
	P3 Amplitude	0.05	0.311	1.000
	P3 Latency	0.02	0.714	1.000
	CTT_InT	0.30	0.000	0.000
	CTT_NInT	0.19	0.014	0.336
Avoidance	N2 Amplitude	0.60	0.276	1.000
	N2 Latency	0.03	0.636	1.000
	P3 Amplitude	0.07	0.130	1.000
	P3 Latency	-0.02	0.686	1.000
	CTT_InT	0.22	0.006	0.144
	CTT_NInT	0.06	0.459	1.000
Arousal	N2 Amplitude	0.18	0.002	0.048
	N2 Latency	0.06	0.274	1.000
	P3 Amplitude	0.13	0.006	0.144
	P3 Latency	-0.07	0.126	1.000
	CTT_InT	0.20	0.011	0.264
	CTT_NInT	0.07	0.396	1.000

Note: Spearman's correlation was used due to non-normality of PCL and Cumulative Trauma Type. Adjusted p-values use a Bonferroni correction.

CTT_InT: Cumulative Interpersonal Trauma Type

CTT_NInT: Cumulative Non-Interpersonal Trauma Type

Table B10. Spearman's Correlation for PCL (and sub-clusters) at post-deployment by ERP Component and Cumulative Trauma.

PCL	Variable	ρ	p-value	adjusted p-value
Overall	N2 Amplitude	0.10	0.119	1.000
	N2 Latency	0.00	0.986	1.000
	P3 Amplitude	0.00	0.944	1.000
	P3 Latency	-0.04	0.390	1.000
	CTT_InT	0.20	0.018	0.432
	CTT_NInT	0.17	0.044	1.000
Re-experiencing	N2 Amplitude	0.13	0.032	0.768
	N2 Latency	0.02	0.732	1.000
	P3 Amplitude	0.08	0.108	1.000
	P3 Latency	-0.07	0.145	1.000
	CTT_InT	0.13	0.132	1.000
	CTT_NInT	0.08	0.322	1.000
Avoidance	N2 Amplitude	0.09	0.149	1.000
	N2 Latency	0.06	0.329	1.000
	P3 Amplitude	-0.06	0.222	1.000
	P3 Latency	-0.06	0.227	1.000
	CTT_InT	0.11	0.189	1.000
	CTT_NInT	0.11	0.175	1.000
Arousal	N2 Amplitude	0.06	0.324	1.000
	N2 Latency	-0.04	0.506	1.000
	P3 Amplitude	0.02	0.664	1.000
	P3 Latency	0.01	0.773	1.000
	CTT_InT	0.18	0.030	0.720
	CTT_NInT	0.17	0.039	0.936

Note: Spearman's correlation was used due to non-normality of PCL and Cumulative Trauma Type. Adjusted p-values use a Bonferroni correction.

CTT_InT: Cumulative Interpersonal Trauma Type

CTT_NInT: Cumulative Non-Interpersonal Trauma Type
